# Bioactive Tryptophan-Based Copper Complex with Auxiliary β-Carboline Spectacle Potential on Human Breast Cancer Cells: In Vitro and In Vivo Studies

**DOI:** 10.3390/molecules26061606

**Published:** 2021-03-14

**Authors:** Walaa Alharbi, Iftekhar Hassan, Rais Ahmad Khan, Shazia Parveen, Khadijah H. Alharbi, Ibtisam I. Bin Sharfan, Ibrahim M. Alhazza, Hossam Ebaid, Ali Alsalme

**Affiliations:** 1 Department of Chemistry, Faculty of Science, King Khalid University, P.O. Box 9004, Abha 62529, Saudi Arabia; 2 Department of Zoology, College of Science, King Saud University, P.O. Box 2455, Riyadh 11451, Saudi Arabia; ihassan@ksu.edu.sa (I.H.); ihazza@ksu.edu.sa (I.M.A.); habdrabou@ksu.edu.sa (H.E.); 3 Department of Chemistry, College of Science, King Saud University, P.O. Box 2455, Riyadh 11451, Saudi Arabia; ibtisam.i.sh@hotmail.com (I.I.B.S.); aalsalme@ksu.edu.sa (A.A.); 4 Chemistry Department, Faculty of Science, Taibah University, Yanbu Branch, 46423 Yanbu, Saudi Arabia; shazia021@gmail.com; 5 Department of Chemistry, Science and Arts College, Rabigh Campus, King Abdulaziz University, Jeddah 21911, Saudi Arabia; khalharbe@kau.edu.sa

**Keywords:** copper complex, MCF7 cells, MTT assay, ROS, comet assay, in vivo toxicity

## Abstract

Biocompatible tryptophan-derived copper (**1**) and zinc (**2**) complexes with norharmane (β-carboline) were designed, synthesized, characterized, and evaluated for the potential anticancer activity in vitro and in vivo. The in vitro cytotoxicity of both complexes **1** and **2** were assessed against two cancerous cells: (human breast cancer) MCF7 and (liver hepatocellular cancer) HepG2 cells with a non-tumorigenic: (human embryonic kidney) HEK293 cells. The results exhibited a potentially decent selectivity of **1** against MCF7 cells with an IC_50_ value of 7.8 ± 0.4 μM compared to **2** (less active, IC_50_ ~ 20 μM). Furthermore, we analyzed the level of glutathione, lipid peroxidation, and visualized ROS generation to get an insight into the mechanistic pathway and witnessed oxidative stress. These in vitro results were ascertained by in vivo experiments, which also supported the free radical-mediated oxidative stress. The comet assay confirmed the oxidative stress that leads to DNA damage. The histopathology of the liver also ascertained the low toxicity of **1**.

## 1. Introduction

Current cancer chemotherapies often fail to improve patient mortality and morbidity due to severe adverse effects on normal tissues. Nowadays, many internal triggers, including pH gradient and enzyme activity, and external stimuli such as light and magnetic field have been integrated with different inorganic and organic materials for a range of biomedical applications such as diagnosis, tissue engineering, and cancer therapy [1].

In 1978, Food and Drug Administration approved cisplatin as an anticancer drug for clinical use. Currently, cisplatin and its analogues are very useful chemotherapeutic agents in treating testicular and ovarian cancers [2]. The modern use of transition metal complexes as chemotherapeutic agents dates back to the serendipitous cisplatin discovery by Rosenberg et al. in 1965 [1,3]. Despite the success of cisplatin and platinum-based drugs, they still stumble upon much resistance, undesirable non-cancer cell toxicity, and limited activity [4,5]. Therefore, the market is always accessible for new advantageous metal-based drugs that offer better viability, such as oral administration, which might diminish severe side-effects and clinic costs. Additionally, research is focused on drugs with higher efficacy, i.e., drugs that interact differently with the target, DNA (deoxyribonucleic acid), can overcome inherent or acquired cisplatin resistance in many tumors, and are active towards tumors non-responsive to current chemotherapy. Several anticancer drugs that could block DNA replication through metal complexes have been developed as DNA binding and/or cleaving agents [6,7].

Copper, a d^9^ metal ion with borderline Lewis acid properties, is considered biologically active, exhibiting unique hydrolytic and redox activities [8]. Therefore, it can bind to different donor atoms present in biomolecules and form complexes with diverse coordination numbers and geometries based on structural versatility, syntheses accessibility, and wide application in various fields. Copper is also an essential cofactor in many enzymes critical for life and a key component in a countless variety of biological functions [9,10,11,12]. Preceding literature on copper complexes discloses copper’s ability to reduce a tumor’s microvascular supply, the tumor volume, and vascular permeability in different forms of cancers [13]. It could be suggested that copper complexes lead to cell death by inducing apoptosis, affecting only cancerous cells while leaving the normal cells unaffected [14,15] and by the generation of reactive oxygen species (ROS) species in oxidative stress, resulting in DNA damage and strand breaks [16].

On the other hand, β-carboline (9*H*-pyrido[3,4-*b*]indole) alkaloids are extensively found in anticancer natural products and are widely distributed in plants, animals, and humans [17,18,19]. They display a broad spectrum of biological and pharmacological activities, including anti-tumor, anti-leishmanial, anti-trypanosomal, anti-HIV, anti-inflammatory, etc. [20,21,22,23,24]. It is widely stated that β-carboline derivatives stimulate anti-tumor and anticancer properties via DNA intercalation [25], inhibition of topoisomerase I and II [26], cyclin-dependent kinase (CDK) [27], and IkK kinase complex (IkK) [28].

In this paper, we report on the synthesis and characterization of ternary Cu(II) (**1**) and Zn(II) complex (**2**) of Schiff base derived from tryptophan and auxiliary β-carboline. Both complexes **1** and **2** were evaluated for their cytotoxicity against MCF7, HepG2, and HEK293 cells by MTT assay, and IC_50_ values were ascertained and compared with the reported literature. The level of LPO, GSH, ROS was studied for the assessment of mechanistic pathways. Both complexes **1** and **2** were further subjected to in vivo studies to evaluate the toxicity to check their suitability as a drug or adjuvant. The tissue samples were subjected to oxidative stress, liver, and renal function markers. Furthermore, histological evaluation and comet assay were also conducted to confirm the biochemical results.

## 2. Results and Discussion

### 2.1. Synthesis and Characterization

**Figure 1:**
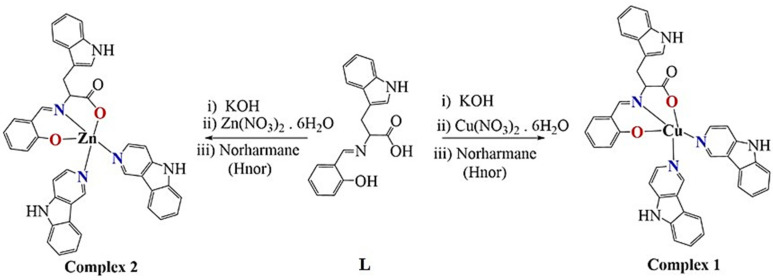
Synthetic route to Schiff base ligand, L and its complexes **1** and **2**.

Two bioactive ligands were mixed to develop new chemotherapeutic entities. The synthesis involved the in situ reaction of tryptophane and salicylaldehyde in the equimolar ratio in methanol to get the characteristic yellow colored solution of Schiff base. TLC (thin layer chromatography) monitored the completion of the reaction. Then added equimolar quantity of metal salts, Cu(II)(NO_3_)_2_·6H_2_O and Zn(II)(NO_3_)_2_·6H_2_O and after 10 min, two folds norharmane (Hnor, β-carboline) to the above solution and allowed to reflux for 4 h (Figure 1). The product obtained was of dark green color Cu(II) complex (**1**) and brownish yellow color Zn(II) complex (**2**) in a significantly good yield of 72–74%. They were soluble in DMSO and DMF, whereas they were partially soluble in H_2_O. Molar conductance revealed non-electrolytic nature [29].

The magnetic moment value of Cu(II) complex **1**, 1.80 μB at 298 K confirms the presence of one electron paramagnetic Cu(II)-d^9^ complex. The DMSO solution’s absorption spectra exhibited π-π*, n-π* transitions at ~285, ~302, ~338, ~354 nm for both the complexes and broad and weak d-d transition ~670 nm for Cu(II) complex **1** possessing square pyramidal geometry ascertained by the reported literature [30,31] and the elemental analysis was found consistent with the proposed structure (Figure S1, Supplementary Materials).

The FT-IR spectra showed a medium intensity peak at ~3418 cm^−1^ for Cu(II) complex **1,** whereas in the case of Zn(II) complex **2**, the peaks got broader and appeared at 3412 cm^−1^ [32]. The broadening could be associated with the coordination of the Hnor ligand with Zn center, a characteristic. The characteristic signal of carboxylate appeared as a strong peak at 1627 cm^−1^ for Cu(II) complex **1**, and 1629 cm^−1^ for Zn(II) complex **2,** which is significantly shifted from the free tryptophan ~1660 cm^−1^ confirms the coordination of the metal center [30]. The characteristic signals of aldimine were displayed at 1600 cm^−1^ for Cu(II) complex **1** and 1597 cm^−1^ for Zn(II) complex **2**. The M-O peak was shown at 552 and 532 cm^−1,^ whereas M-N signals were observed at ~425 cm^−1^ (Figure S2, Supplementary Materials).

The ^1^H NMR spectrum for proton in DMSO-d_6_ of the Zn(II) complex **2** displayed all the characteristic peaks associated with the structure proposed (Figures S3 and S4, Supplementary Materials). The signal related to Hnor N-H appeared at 11.96 ppm, whereas the N-H of the tryptophane moiety appeared at 10.85, as characteristic singlet and broad. The distinct singlets for the Hnor proton peak are attributed at 8.98 and 8.37 ppm. The other aromatic protons peaks appeared at 8.23–8.10, 7.63–7.51, 7.29–7.21, 7.04–6.90 ppm (multiplets), and 6.62, 6.46, 6.20 ppm (singlets). The aliphatic protons peaks exhibited at 3.32, 2.06, 1.77 ppm for the tryptophan moiety. The slight broadening of the NMR signals is endorsed to coordinate with the Zn(II) metal center (Figure 1).

Both the Cu(II) complex **1** and Zn(II) complex **2** exhibited stability in solution as evidenced by principally demonstrating molecular ion peaks [M + nH]^+^ in ESI-MS in DMSO [31]. The emission properties of Cu(II) complex **1** and Zn(II) complex **2** in DMSO exhibited a band at ~360 nm, ~390 nm, and an extra peak in Zn(II) complex **2** at 450 nm (characteristic of zinc complex **2**) (Figure S5, Supplementary Materials).

### 2.2. Computational Studies: Density Functional Theory

**Figure 2:**
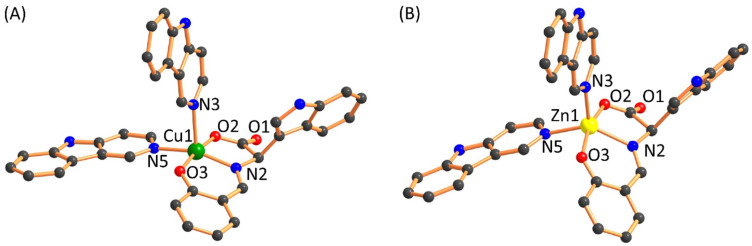
DFT optimized structures of complex (**A**) **1** and (**B**) **2**. Only coordinated donor atoms are labeled. Hydrogen atoms are omitted for clarity.

Density function theory (DFT) calculations were performed to investigate the geometric and electronic features, as the crystal structures of complexes have not been obtained. The proposed structures have been optimized with the B3LYP level of DFT. The optimized structures of both the complexes are shown in Figure 2, indicating the geometry around the d^9^ Cu(II), and d^10^ Zn(II) ion are found to be distorted square pyramidal with the two donor nitrogen atom of the norharmane ligand and one nitrogen and two oxygen donor atoms of the Schiff base ligand moiety. The calculated bond lengths are given in Table 1 and are in good agreement with the previously reported single-crystal X-ray data in various papers.

The vibrational spectra have also been simulated to validate complexes’ proposed structure (Figure S6, Supplementary Materials). The calculated frequencies and other spectral features were found within the range shown in Table 2. Three factors could be responsible for the deviation in the computed spectra, (1) the environmental aspect as DFT calculations were performed with solvation effect (liquid phase) while experimental data was obtained at solid-state; (2) the calculated frequencies are contained only harmonic while experimental have both harmonic and anharmonic effect; and (3) basis set discrepancies. However, the pattern and trend of spectra were quite similar in both cases, which validate the proposed structures for complexes.

Moreover, we have also calculated the UV-vis spectra to support further the complexes’ calculated geometry (Figure S7, Supplementary Materials). The TDDFT (time-dependent density functional theory) calculations have been performed with the DMSO as a solvation effect for both the complexes. The significant features of the calculated UV–vis spectra are in good match with experimental spectra. Interestingly, the experimentally observed band at ~670 nm range also observed in TDDFT calculated spectrum, which is absent in the complex **2**, alternatively validated the complexes’ proposed molecular geometry.

**Figure 3:**
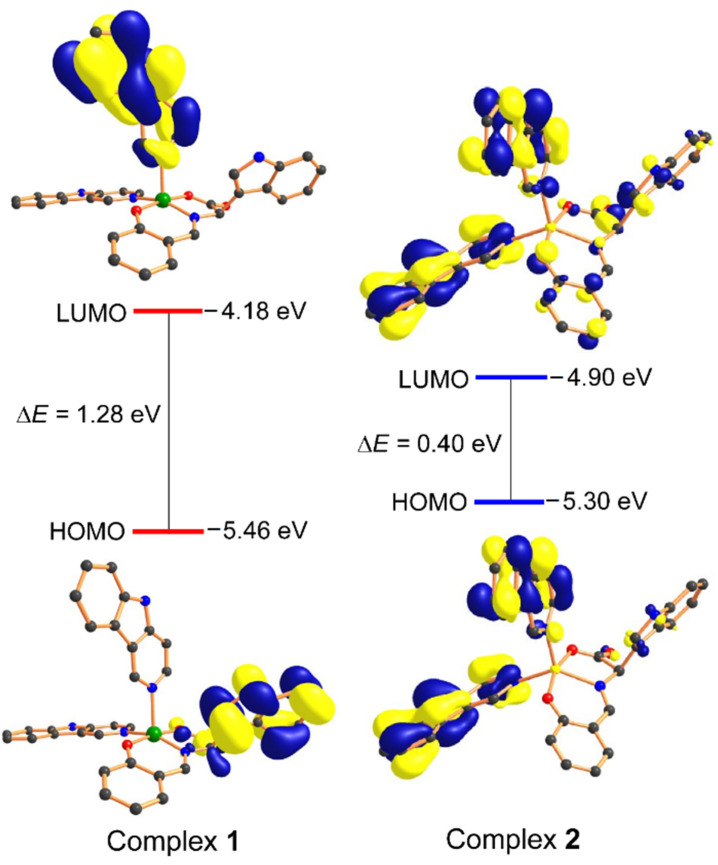
Frontier molecular orbitals of complexes **1** and **2** and their HOMO-LUMO energy gaps.

Literature reveals that the HOMO (highest occupied molecular orbital) and LUMO (lowest unoccupied molecular orbital) energy parameters could be related to the molecules’ biological activities. A small energy gap (ΔE) between the HOMO and LUMO indicates more polarizable molecule behavior and acts as a soft molecule with higher chemical and biological activity. However, molecules with a more significant energy gap offer more excellent stability and lower activity than those with smaller HOMO-LUMO energy gaps. The HOMO of complex **1** was mainly localized on the ‘Schiff base ligand’ moiety while the LUMO on the ‘Hnor’ ligand. Whereas HOMO of complex **2** is found primarily on the ‘Hnor’ ligand and LUMO delocalized on the whole molecule. Interestingly, the HOMO-LUMO energy gap of complex **1** (1.28 eV) is larger than the complex **2** (0.40 eV), suggested that complex **2** could show more significant biological activity as compared to complex **1** (Figure 3).

### 2.3. In-Vitro Anticancer Activity

#### 2.3.1. MTT Assay

The cytotoxicity of the synthesized complexes **1** and **2** were studied by treating against the human cancer cell lines, MCF7 (breast) and HepG2 (hepatocellular), and compared with HEK293 (non-tumorigenic cells). The MTT assay was performed to obtained IC_50_ values for complexes **1** and **2**. The significance of the IC_50_ value of complexes **1** and **2** were compared with the standard drug, cisplatin, copper, zinc nitrate salts, and the previously reported compound to study the structure–activity relationship. The results exhibited significantly good activity of complex **1** against MCF7 cancer cells compared to previously reported complexes of copper derived from tryptophan, and diamine with non-Schiff’s base, Schiff’s base and reduced Schiff base (Table 3).

#### 2.3.2. Morphology of the MCF7 Cancer Cells

**Figure 4:**
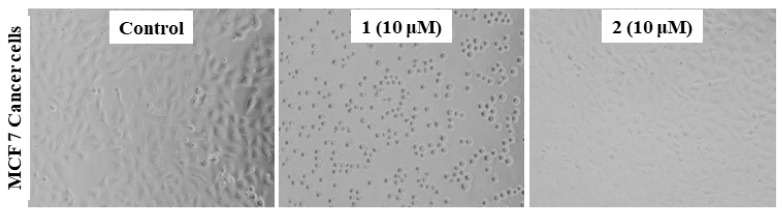
Morphology of the human breast cancer (MCF7) cells, untreated (control) and treated with Cu(II) (**1**) and Zn(II) (**2**) complexes.

One of the preliminary investigations is studying the morphology of the cancer cells before and after treatment. Thus, we examined the changes in MCF7 cancer cells’ morphology upon treatment with complex **1** and **2** at a concentration of 10 μM. The results displayed a significant reduction in the cell adhesion capacity, a loss of its characteristic feature compared to when treated with **2**, and the untreated (control) (Figure 4). This loss to the cell’s adhesion capacity may be associated with the penetration of **1** into the cells and enable interaction with key biomolecules inside, such as DNA and HSA (human serum albumin). This motivated us to study complex **1** more in detail, and we have explored other parameters, *viz.,* changes in the level of glutathione, lipid peroxidation, and ROS generation.

#### 2.3.3. Assessment of the Oxidative Stress against MCF7 Cells

**Figure 5:**
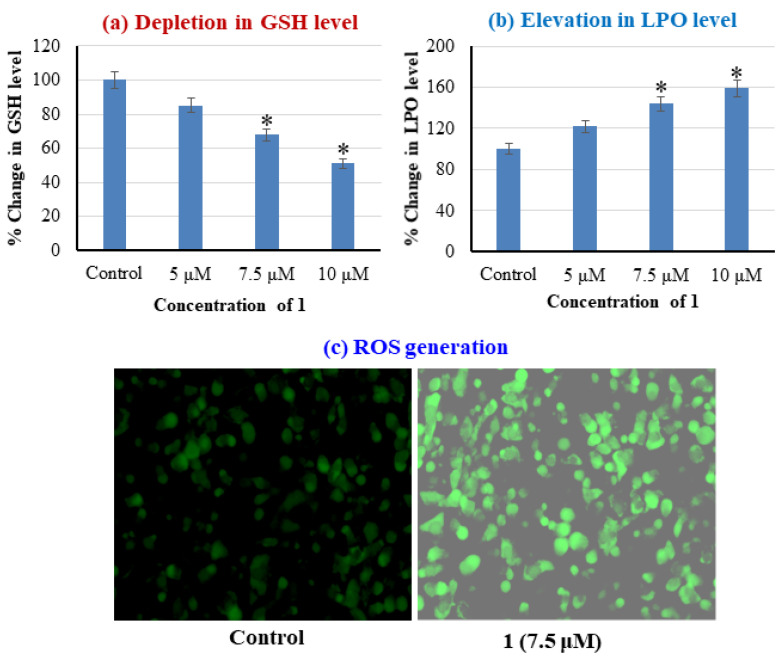
(**a**) Glutathione level in MCF7 cell line after the exposure of complex **1** for 24 h at 5, 7.5, and 10 µM concentrations. (**b**) Induction in LPO level in MCF7 cell line after the exposure of complex **1** for 24 h at 5, 7.5, and 10 μM concentrations. (**c**) ROS generation in MCF7 cells exposed to complex **1** for 24 h at 7.5 μM concentration. * indicates statistically different from the control.

The mechanism of action cytotoxicity of complex **1** against MCF cells was assessed by studying the oxidative stress via examining the levels of GSH (Glutathione), LPO (Lipid peroxidation), and visualizing ROS generation. GSH is one of the plentiful thiol compounds in the human body [40,41,42]. The disulfide bridged GSH molecules *viz.,* GSSH (oxidized GSH), and GSH (free) exist in equilibrium. When the ratio of GSH/GSSH > 10, this depletion is often associated with cancer via affection regulation of cell cycle, DNA synthesis, mutations, etc. [43,44]. It is also well established that the concentration of GSH is higher in cancer cells than in normal cells [43,45]. The GSH reduced or oxidized form is known to form adducts with Cu(II)/(I) and exhibits an antioxidant and superoxide dismutation property [37]. Therefore, we evaluated the impact of 1 on the intracellular level of GSH in MCF7 cancer cells. The results revealed in MCF7 cells treating with complex **1** decreases the level of GSH in a concentration dependent manner. At 5 μM (lower than IC_50_) of complex **1**, the depletion is ~15%, whereas, at ~IC_50_ value ~7.5 μM, it showed a significant reduction of 32% and above IC_50_, 10 μM is ~49% (Figure 5a).

The integrity of the cell membranes and the organelles are known to get disturbed by LPO caused by lipids oxidative damage. It is kinetically and thermodynamically driven, i.e., peroxyl (H_2_O•)/superoxide (O_2_•^−^) radicals swiftly react with lipid/lipid radicals, respectively. Hence the assessment of the LPO is one of the vital parameters. The results of **1** against MCF7 cells LPO level exhibited a significant elevation of ~44% at ~IC_50_ value. The sub-IC_50_ values of 5 μM demonstrated ~22%, and above IC_50_, 10 μM showed an increase of ~59% (Figure 5b).

The production of ROS generation was also visualized as it well established that copper-based potential cancer chemotherapeutics give rise to OH•, ^1^O_2_•, and O_2_•^−^ radicals. The ROS production is associated with copper irrespective of the oxidation state +2/+1 inside cells.

Cu(I) reduces H_2_O_2_ generate OH•, Cu(II) reduced to Cu(I) by O_2_•^−^/GSH [46,47]. The presence of surplus intracellular ROS causes DNA damage and may trigger apoptotic p53 gene with other genes. Thus, it is interesting to explore the ROS generation in MCF cancer cells by treating it with complex **1**. The results upon treatment of MCF7 with **1** displayed the significant ROS generation. Cells images are presented in Figure 5c, indicating green fluorescent cells ascertaining the substantial ROS generation compared to control (untreated). The elevation of ROS, LPO level, and depletion of GSH level in the MCF7 cancer cell, upon treatment with complex **1** ascertains the oxidative DNA damage as a possible mode of action. These results are in accordance with the literature reported [37,48].

### 2.4. In Vivo Studies

In vivo study was conducted to investigate if any serious toxic issues are associated with the novel complexes in reference to an established toxicant, CCl_4_ (carbon tetrachloride), in vivo. For this, we assessed the liver and kidney function markers in the serum samples. The involvement of free radicals mediated oxidative stress observed in vitro study on cell lines was also checked in vivo by measuring the reduced glutathione (GSH) and MDA (malondialdehyde) levels in the liver tissue samples. Furthermore, these biochemical parameters were confirmed by histological analysis and comet assay of the liver samples from the treated groups.

#### 2.4.1. Effect on Liver Function Markers

**Figure 6:**
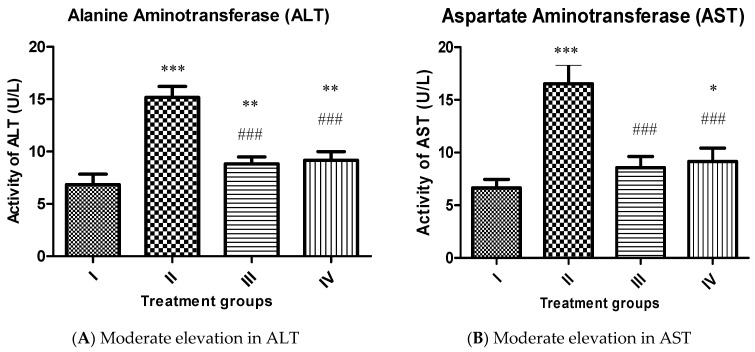
Showing bars of liver function markers (ALT (**A**) and AST (**B**)) and of the indicated groups. All the data has been expressed in mean ± SEM for each group. * indicates statistically different from the control (group I) at *p* ≤ 0.05. Also ** indicate *p* ≤ 0.005 while *** and ### indicate *p* ≤ 0.001.

Alanine aminotransferase (ALT):

Group II exhibited a rise in its activity by 121.92% compared to the control, group I confirming extensive live damage. However, group III and IV showed decreased liver markers by 41.96% and 39.59% concerning group II (Figure 6A).

Aspartate aminotransferase (AST):

Group II demonstrated an increase in its activity by 148.49% regarding group I whereas, group III and IV exhibited a decrease in its activity by 48.21% and 44.65% compared to group II (Figure 6B).

#### 2.4.2. Effect on Renal Function Markers

Urea and creatinine are chosen for the assessment of toxic insults on kidney function in the present study.

**Figure 7:**
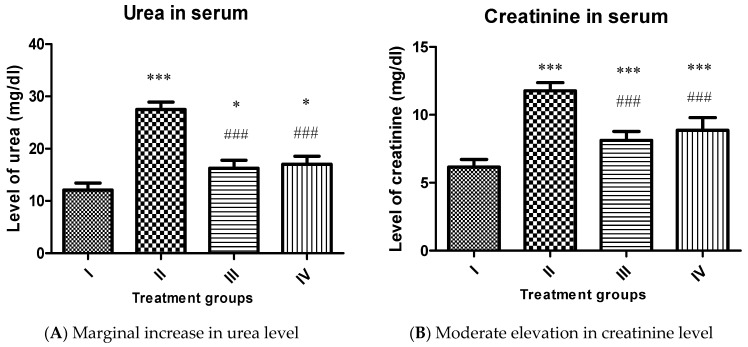
Showing bars of renal function markers (Urea (**A**) and Creatinine (**B**)) of the indicated groups. All the data has been expressed in mean ± SEM for each group. * indicates statistically different from the control (group I) at *p* ≤ 0.05. Also *** and ### indicate *p* ≤ 0.001.

Urea:

A similar pattern was observed in the urea level in the treated groups. Group II showed enhancement in its level by 127.40%, while group III and IV exhibited a decline in its level by 40.80% and 38.04% concerning group II (Figure 7A).

Creatinine:

In the present study, the level of creatinine was elevated by 91.38% in group II compared to the control (group I). However, group III and IV showed a decline in its level by 31.01% and 24.72%, respectively, compared to group II (Figure 7B). It is evident from the pattern of the liver and renal function markers that both complexes induce a certain extent of damage in the liver and kidney of the treated animals. Yet, their toxicity is significantly less as compared to CCl_4_. Our results are in accordance with the previously reported [49,50,51]. This indicates that the proposed complexes are suitable for in vivo administration in moderation. However, it is noteworthy that complex **1** was less toxic than complex **2** in vivo.

#### 2.4.3. Effect on Key Antioxidant Parameters

After evaluation of toxicity of the complexes, we wanted to investigate if free-radicals mediated oxidative stress is triggered post administration of the complexes in vivo. To confirm, the level of GSH and MDA was estimated in the target organ-liver tissue samples.

**Figure 8:**
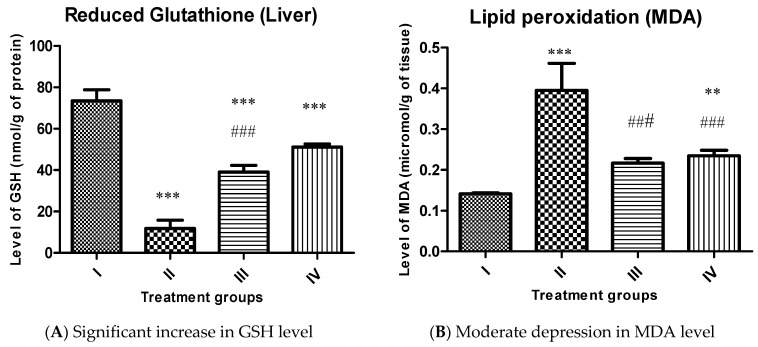
Showing bars of oxidative stress markers (GSH Urea (**A**) and MDA Urea (**B**) levels) of the indicated groups. All the data has been expressed in mean ± SEM for each group. ** indicate *p* ≤ 0.005 while *** and ### indicate *p* ≤ 0.001.

Reduced glutathione level (GSH):

It is considered one of the most antioxidant markers to assess the burden of oxidative stress in vivo. Group II showed almost 84% of the decline in its level compared to group I, while group III and IV also exhibited an increase by 231.77% and 333.38% compared to group II (Figure 8A).

Malondialdehyde (MDA):

It is assumed to be also a very reliable marker to assess the extent of LPO following oxidative insult in vivo. Group II showed a staggeringly enhanced MDA level by 180.14%, while group III and IV demonstrated a decrease in its level by 45.06% and 40.75% with respect to group II (Figure 8B).

The present study shows that both the complexes improve the antioxidant status in vivo as the GSH level is enhanced, and a marked decline in MDA in CCl4 challenged rats after treating complexes. It is evitable that both Cu and Zn increase the Cu-Zn SOD activity and assist in the absorption and digestion of the food in the treated animals [52]. The results imply that both the complexes perturb the redox balance in the target organ compared to the control. It is surprising to see that complex **2,** despite generating a higher amount of MDA, replenishes more GSH concerning complex **1**.

#### 2.4.4. Comet Assay

**Figure 9:**
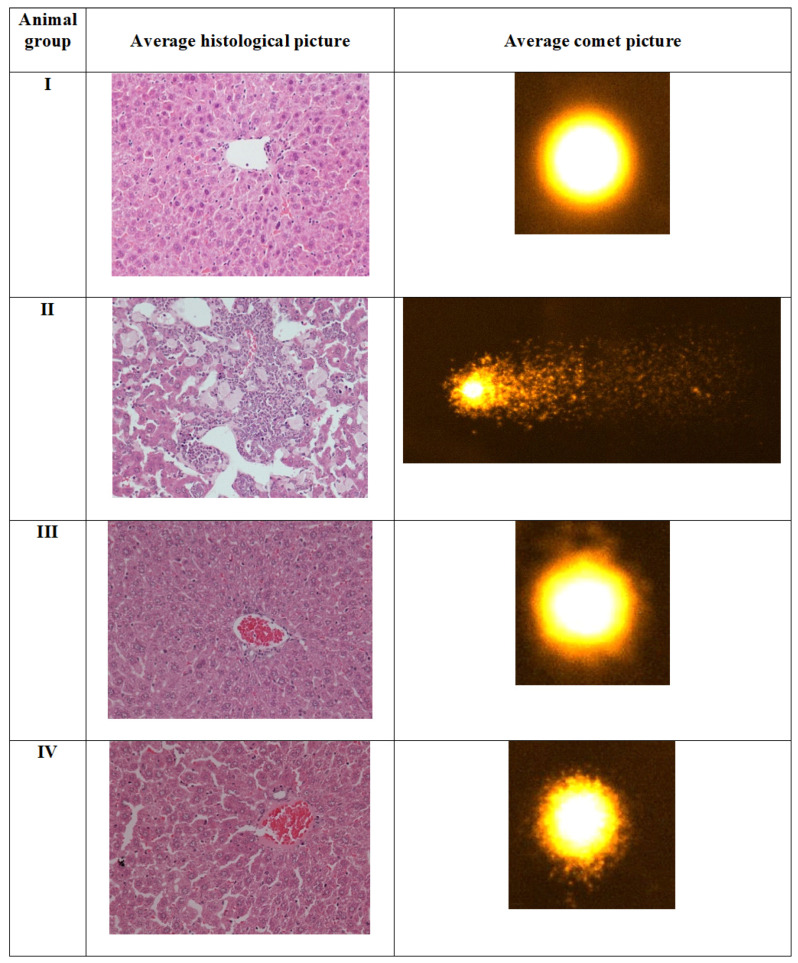
Average histological photomicrographs and Comet assay performed on the indicated groups. Group **I** shows the control without any treatment, while group **II** is control positive treated with CCl_4_. Group **III** and **IV** are complex **1** and **2** respectively.

This is a susceptible technique to assess nuclear damage following any chemical/compound treatment in the animals. In the present investigation, group II (CCl_4_ treated) demonstrated an increase in comet tail-length by 156.40% in the liver samples than group I, as previously reported [50]. The genotoxicity of CCl_4_ is well established by comet assay [49,53]. However, group III and IV showed decreased tail-length by 42.68% and 39.08% concerning group II (Figure 9). Hence, both the complexes show the protective efficacy against the toxicant-induced damage in the target tissues’ nuclear DNA. It is noteworthy that complex **1** showed marginally better protective efficacy as compared to complex **2**.

### 2.5. Histopathological Evaluation in the Liver Samples

The histomicrographs of Group II demonstrated vivid features of acute toxicity in the form of inflammation and extensive tissue damage (Figure 9). The sinusoids were abruptly disturbed, showing that tissue fibrosis and nuclear damage were in most of the hepatocytes, as previously documented [51,54]. The sections from this group demonstrated typical liver injury evidenced by noticeable macrosteatosis, ballooning of several hepatocytes, loss of cytoplasm, and pushing of nuclei towards one side, depicting the cellular necrosis. Group III and IV showed mild toxic insults with mostly hepatocytes maintain their microstructure in the sections; however, moderate inflammation features and tissue haemorrhage were observed. These features were less in group III than IV, entailing that the complex **1** elicited less toxic insults in the target organs. The in vivo study was aimed to investigate the suitability of usage of the novel Cu(II) (**1**) and Zn(II) (**2**) complexes to be exploited as a drug or drug adjuvant in vivo. CCl_4_ is an established hepatotoxicant to assess comparative toxicity in animal-based studies in many previous investigations [51,55]. In vivo results of this study show that CCl_4_ caused severe toxicity in the treated animals’ liver. Both complexes were well tolerated in the animals as data for the studied parameters were comparable to the CN^−^. The investigation also reveals that the complexes affect the liver more than the kidneys in the treated rodents. Complex 1 was more suitable as a drug or drug adjuvant in vivo than complex **2**.

## 3. Materials and Methods

### 3.1. Chemicals and Instrumentation

All chemicals and solvents were purchased from commercial sources like Sigma-Aldrich and Fluka (Taufkirchen, Germany) and were used as received. Instruments utilized Perkin-Elmer 2400 Series II CHNS/O elemental analyzer for analyses (Ohio, USA), Shimadzu IR-Affinity spectrophotometer for FTIR (4000–400 cm^−1^), CARY 100 Bio VARIAN UV-vis spectrophotometer, JEOL-ECP-400 spectrometer (NMR), Melting points were determined on a BUCHI Melting point B-540, Sherwood Scientific Magnetic Balance MSB Auto. The kits from QCA and Linear diagnostic kits (Spain) are utilized for biochemical analysis.

### 3.2. Synthesis

#### 3.2.1. Synthesis of [Cu(L)(Hnor)_2_] Complex (**1**)

The copper complex was prepared by the addition of tryptophan (200 mg, 1.0 mmol), NaOH (40 mg, 1.0 mmol), salicylaldehyde (0.125 mL, 1.0 mmol) in a 1:1:1 ratio in dry methanol as reported earlier [31]. After completing the reaction monitored with the thin layer chromatography (TLC), the Cu(NO_3_)_2_·6H_2_O (241 mg, 1.0 mmol) was added. The norharmane ‘Hnor’ (336 mg, 2.0 mmol) was added dropwise for 20 min to the above reaction mixture. Then, stirred at 70 °C for 4 h. Then the solution mixture was cooled to room temperature and filtered, and left for slow evaporation. The dark green color crystalline product was then isolated. Yield: 74%. Anal. Calcd for C_40_H_30_N_6_O_3_Cu (%): C, 68.03; H, 4.28; N, 11.90. Found: C, 67.97; H, 4.26; N, 11.89. FT-IR (KBr pellet, cm^−1^): 3418, 3044, 1627, 1601, 1536, 1496, 1448, 1350, 1244, 1147, 1087, 1047, 730, 565, 552, 477, 444, 425. ESI-MS (DMSO): *m*/*z* for C_40_H_30_N_6_O_3_Cu+H^+^: 706.2 [M + H]^+^. μ_eff_ (298K): 1.80 μB.

#### 3.2.2. Synthesis of [Zn(L)(Hnor)_2_] Complex (**2**)

The zinc complex was synthesized by using the above protocol using the Zn(NO_3_)_2_·6H_2_O (297 mg, 1.0 mmol). Yield: 72%. Anal. Calcd for C_40_H_30_N_6_O_3_Cu (%): C, 67.85; H, 4.27; N, 11.87. Found: C, 67.82; H, 4.27; N, 11.84. FT-IR (KBr pellet, cm^−1^): 3412, 3052, 1629, 1597, 1540, 1500, 1448, 1331, 1247, 1147, 1082, 10,444, 733, 633, 532, 470, 424. ESI-MS (DMSO) *m*/*z* for C_40_H_30_N_6_O_3_Zn+2H^+^: 710.0 [M + H]^+^. ^1^H NMR (400MHz, DMSO-*d_6_*, δ, ppm): 11.96 (s, 2NH, Hnor), 10.85 (s,1NH, tryptophan), 8.98 (s, 2CH, Hnor), 8.37 (s, 2CH, Hnor), 8.23–8.10 (m, 3H, 2H-Hnor, 1H-H-C=N), 7.63–7.51 (m, 4H, 2H-Hnor, 1H-tryptophan, 1H-aldehyde), 7.29–7.21 (m, 5H, 4H-Hnor, 1H-Aldehyde), 7.04–6.90 (m, 5H, 2H-Hnor, 2H-tryptophan, 1H-aldehyde), 6.62 (s, 1H, tryptophan), 6.46 (s, 1H, tryptophan), 6.20 (s, 1H, aldehyde), 3.32 (m, 1H-tryptophan), (2.06 (m, 1H-tryptophan), 1.77 (m,1H-tryptophan).

### 3.3. Computational Methodology

The full geometry optimization, single point energy, vibrational frequency analysis and the TDDFT (time-dependent density functional theory) calculations have been carried out at DFT level of theory using the B3LYP functional [56,57,58] with the help of the Gaussian-09 program package [59]. The calculations were performed using 6–31G* basis sets [60,61] for C, H, N, O, S atoms and typical effective core potential (ECP) basis LanL2DZ (Los Alamos National Laboratory 2 double ζ) as extra basis set [62] for copper and zinc atoms. All the DFT calculations were performed without counter ions by employing the polarizable continuum model, CPCM (DMSO as solvent) [63,64,65]. No symmetry restrictions have been applied during geometry optimization. The Hessian matrix was calculated analytically for the optimized structures in order to prove the location of correct minima (no imaginary frequencies). The Cartesian atomic coordinates of the calculated optimized structures in DMSO are given in Table S1 (Supplementary Materials).

### 3.4. In Vitro Cytotoxicity

All the experiments were carried out using standard protocols [43,45,47,66,67,68,69,70,71,72] and slight modification adopted by us [73,74,75,76,77,78,79,80,81]. Cell culture of HepG2 and MCF7 cancer cell lines were grown in DMEM and maintained at 37 °C. MTT assay was carried out and read at 550 nm, and the IC_50_ value was evaluated. Morphological images were taken on a phase-contrast microscope at 20× magnification. The generation of ROS was assessed by DCFH-DA (2ʹ,7ʹ-Dichlorofluorescin diacetate) dye as per the protocol, and images were taken using a fluorescence microscope. The Chandra et al. protocol carried out the intracellular GSH depletion and read the absorbance at 412 nm [76]. The LPO assay was performed using TBARS (thiobarbituric acid reactive substances) protocol, and the absorbance of the supernatant was read at 550 nm.

### 3.5. In Vivo Toxicity Profiling

After confirming the antineoplastic efficacy of the complexes in the cell lines, we desired to check if they are suitable for administration in the animals and how much toxicity they can elicit in vivo in reference of the established toxicant, CCl_4_. In this section, we tried to explore the effects of the complexes on the liver and kidney as both are the key organs of xenobiotic metabolism, i.e., to assess the toxicity of any new chemical or substance in vivo.

#### 3.5.1. Animal Treatment and Sample Preparation

Thirty-six adult Swiss albino rats (120–140 g, 6–8 weeks old) were purchased from the central animal house, Department of Pharmacy, King Saud University, Riyadh, KSA. They were housed and taken care of as previously done in the departmental animal house in the controlled conditions [82]. All the animals were randomly divided into four groups (*n* = 6) as follows:

Group I: Control normal without any treatment.

Group II (Control positive): Rats treated with CCl_4_ with a single dose of 1 mL/kg in liquid paraffin in the ratio of 1:1 by volume.

Group III: Rats were injected with the synthetic Complexes **1** once a week at the dose of 1 mg/kg body weight for one month.

Group IV: Rats were injected with the synthetic Complexes **2** once a week at the dose of 1 mg/kg body weight for one month.

All the animals were injected these test chemicals intraperitoneally with 1 mL syringe (BD Science, USA). Both the complexes were chosen to assess their efficacy against CCl_4_ induced hepatotoxicity in vivo [51]. This in vivo study was aimed to investigate if the complexes cause any substantial toxicity in the animals after their repeated dose [83]. Their serum and liver were stored for basic biochemical analysis that were further confirmed by histopathology and comet assay.

All the animal-based experiments were conducted in accordance with the standards set forth under the guidelines for the care and use of experimental animals by the Committee for the Purpose of Control and Supervision of Experiments on Animals and the National Institutes of Health. The treatment method and study protocol (care and handling of experimental animals) were approved by the Animal Ethics Committee of the Zoology Department in the College of Science at King Saud University, Riyadh (KSA). The liver and serum samples were prepared for biochemical analysis as previously mentioned [84].

#### 3.5.2. Estimation of Aspartate Aminotransferase (AST) and Alanine Aminotransferase (ALT) as Liver Function Markers

For assessment of toxicity of the complexes on the functionality of the organ, the established markers (AST and ALT) were estimated in the serum sample by commercially available estimation kits (QCA, Spain) under the manufacturer’s procedural instructions.

#### 3.5.3. Measurement of Reduced Glutathione (GSH) and Malondialdehyde (MDA)

For the assessment of involvement of free radicals after administration of the complexes, level of GSH, and lipid peroxidation (LPO or MDA) were measured in the liver tissue samples. The level of reduced glutathione (GSH) was estimated by the method of Jollow et al. (1974) [84], while the measurement of total malondialdehyde (MDA) was estimated by the method of Beuge and Aust (1978) [77].

#### 3.5.4. Comet Assay

Furthermore, we were interested to know if the complexes affect the integrity of the nuclear DNA in the treated animals. We executed a comet assay of the liver cell suspension as per the previously established method with slight modifications [85,86].

#### 3.5.5. Histopathological Assessment of Liver Tissues

All the biochemical findings were cross-checked with the histological evaluation of the liver tissues. The processing of the tissue, staining, and snapping were conducted as previously done [52].

#### 3.5.6. Statistical Analysis

The data generated during the present work was evaluated by one-way ANOVA, followed by post-hoc analysis by Tukey’s method using Graph Pad Prism 5 software. All the data has been expressed in mean ± SEM for each group. * indicates statistically different from the control (group I) at *p* ≤ 0.05 while # indicates statistically different control positive (group II). ** and ## indicate *p* ≤ 0.005 while *** and ### indicate *p* ≤ 0.001.

## 4. Conclusions

New biocompatible ternary Cu(II) **1** and Zn(II) **2** complexes bearing β-carboline and tryptophan were synthesized and characterized. The validation of the structures of the complexes **1** and **2** was performed by a computational method using TD-DFT calculation of IR and UV-vis absorption. These complexes were tested for cytotoxicity against two cancer cell lines, MCF7, HepG2, and a non-tumorigenic cell line, HEK293. The results exhibited significantly good activity of **1** against MCF7 cells. Then, complex **1** was studied for cytotoxicity mechanistic pathway in vitro, in which the level of GSH reduced and LPO get elevated to a significant level and gave rise to ROS generation.

Furthermore, in vivo experiments also ascertained the ROS generation. Comet assay performed showed the oxidative damage of DNA, which is consistent with the above results. The liver and kidney function marker studied approves the in vivo administration (in moderation) of the complexes and confirmed the low toxicity of complex **1**. Thus, complex **1** exhibits the potential to act as an anticancer drug and warrants further investigations.

## Figures and Tables

**Table 1:** Selected bond distances (Å) for complexes **1** and **2**.

Bonds	1 (Calculated)	2 (Calculated)	1 (Experimental)	2 (Experimental)	References
Cu(1)-N(2)/Zn(1)-N(2)	1.99	2.136	~1.981–2.015	~2.000–2.280	[31,33,34,35]
Cu(1)-N(3)/Zn(1)-N(3)	2.346	2.134			
Cu(1)-N(5)/Zn(1)-N(5)	2.116	2.161			
Cu(1)-O(2)/Zn(1)-O(2)	1.984	2.061	~1.952–2.325	~1.990–2.089	[31,33,34,36]
Cu(1)-O(3)/Zn(1)-O(3)	1.958	2.069			

**Table 2:** Some selected experimental and calculated frequencies (cm^−1^) of complexes **1** and **2**.

Vibrational Band	Complex 1 (Experimental)	Complex 1 (Calculated)	Complex 2 (Experimental)	Complex 2 (Calculated)
ν(N-H) stretching	3418	3509	3412	3502
ν(N=CH) stretching	1601	1610	1597	1609
ν(-O˗C=O) stretching	1627	1656	1629	1653

**Table 3:** Cell viability assay (MTT assay) gives the IC_50_ values (µM) to treat two human cancer cell lines (HepG2 and MCF7) and a non-tumorigenic cell (HEK293).

Complex	HepG2 (µM)	MCF7 (µM)	HEK293 (µM)	References
[Cu(L)(Hnor)_2_] (**1**)	21.7 ± 0.9	7.8 ± 0.4	>100	This work
[Zn(L)(Hnor)_2_] (**2**)	50 ± 1.5	55 ± 1.9	>200	This work
[Cu(tryp) (Hnor)_2_]	27.0 ± 1.1	10 ± 1.3	>100	[37]
[Zn(tryp)(Hnor)_2_]	88 ± 1.9	24 ± 1.7	>150	[37]
[Cu(Sal-Trp)(dppz)]	ND	32.56 ± 3.05	ND	[31]
[Cu(Sal-Trp)(nip)]	ND	20.33 ± 5.50	ND	[31]
Cisplatin	7.63 ± 1.6	38 ± 1.23	>50	[38,39]
Cu(NO_3_)_2_	>200	>200	>200	[37]
Zn(NO_3_)_2_	>200	>200	>200	[37]
Hnor	>200	>200	>200	This work
L	>200	>200	>200	This work

## Data Availability

Data is contained within the article or supplementary material.

## Supplementary Materials

The following are available online. Absorbance spectra of complexes **1** and **2**, Figure S2: IR spectra of complexes **1** and **2**, Figure S3: ^1^H NMR of Zn complex, **2**, Figure S4: ^1^H NMR of Zn complex, **2** (aromatic region), Figure S5: Fluorescence spectra of complexes **1** and **2**, Figure S6: DFT calculated vibrational spectra of complex **1** (blue) and **2** (red), Figure S7: TDDFT calculated electronic absorption spectra of complex **1** (red) and **2** (blue). Table S1: The Cartesian atomic coordinates of the calculated optimized structures **1** and **2** in DMSO.

## Sample Availability

Samples of the compounds are available from the authors.

## References

[1] Parveen S., Arjmand F., Tabassum S. (2019). Clinical developments of antitumor polymer therapeutics. RSC Adv..

[2] Lippert B. (2006). Cisplatin: Chemistry and Biochemistry of a Leading Anticancer Drug.

[3] Rosenberg B., Van Camp L., Krigas T. (1965). Inhibition of cell division in Escherichia coli by electrolysis products from a platinum electrode [17]. Nature.

[4] Wang X., Wang X., Guo Z. (2015). Functionalization of Platinum Complexes for Biomedical Applications. Acc. Chem. Res..

[5] Johnstone T.C., Suntharalingam K., Lippard S.J. (2016). The next generation of platinum drugs: Targeted Pt (II) agents, nanoparticle delivery, and Pt (IV) prodrugs. Chem. Rev..

[6] Dasari S., Bernard Tchounwou P. (2014). Cisplatin in cancer therapy: Molecular mechanisms of action. Eur. J. Pharmacol..

[7] Santini C., Pellei M., Gandin V., Porchia M., Tisato F., Marzano C. (2014). Advances in copper complexes as anticancer agents. Chem. Rev..

[8] Pearson R.G. (1963). Hard and Soft Acids and Bases. J. Am. Chem. Soc..

[9] Vella F. (1995). Principles of Bioinorganic Chemistry.

[10] Holm R.H., Kennepohl P., Solomon E.I. (1996). Structural and functional aspects of metal sites in biology. Chem. Rev..

[11] Metzler-Nolte N., Kraatz H.B. (2007). Concepts and Models in Bioinorganic Chemistry.

[12] Solomon E.I., Heppner D.E., Johnston E.M., Ginsbach J.W., Cirera J., Qayyum M., Kieber-Emmons M.T., Kjaergaard C.H., Hadt R.G., Tian L. (2014). Copper active sites in biology. Chem. Rev..

[13] Denoyer D., Masaldan S., La Fontaine S., Cater M.A. (2015). Targeting copper in cancer therapy: “Copper That Cancer”. Metallomics.

[14] Tardito S., Bassanetti I., Bignardi C., Elviri L., Tegoni M., Mucchino C., Bussolati O., Franchi-Gazzola R., Marchiò L. (2011). Copper binding agents acting as copper ionophores lead to caspase inhibition and paraptotic cell death in human cancer cells. J. Am. Chem. Soc..

[15] Zuo J., Bi C., Fan Y., Buac D., Nardon C., Daniel K.G., Dou Q.P. (2013). Cellular and computational studies of proteasome inhibition and apoptosis induction in human cancer cells by amino acid Schiff base-copper complexes. J. Inorg. Biochem..

[16] Ng C.H., Kong S.M., Tiong Y.L., Maah M.J., Sukram N., Ahmad M., Khoo A.S.B. (2014). Selective anticancer copper(ii)-mixed ligand complexes: Targeting of ROS and proteasomes. Metallomics.

[17] Siegel R., Naishadham D., Jemal A. (2013). Cancer statistics, 2013. CA Cancer J. Clin..

[18] Haskel C.M., Haskell C.M. (2001). Breast cancer. Cancer Treatment.

[19] Belluti F., Fontana G., Bo L.D., Carenini N., Giommarelli C., Zunino F. (2010). Design, synthesis and anticancer activities of stilbene-coumarin hybrid compounds: Identification of novel proapoptotic agents. Bioorganic Med. Chem..

[20] Brummond K.M., Goodell J.R., La Porte M.G., Wang L., Xie X.Q. (2012). Synthesis and in silico screening of a library of β-carboline-containing compounds. Beilstein J. Org. Chem..

[21] Ikeda R., Kimura T., Tsutsumi T., Tamura S., Sakai N., Konakahara T. (2012). Structure-activity relationship in the antitumor activity of 6-, 8- or 6,8-substituted 3-benzylamino-β-carboline derivatives. Bioorg. Med. Chem. Lett..

[22] Lunagariya N.A., Gohil V.M., Kushwah V., Neelagiri S., Jain S., Singh S., Bhutani K.K. (2016). Design, synthesis and biological evaluation of 1,3,6-trisubstituted β-carboline derivatives for cytotoxic and anti-leishmanial potential. Bioorganic Med. Chem. Lett..

[23] Silva C.M.B.L., Garcia F.P., Da Silva Rodrigues J.H., Nakamura C.V., Ueda-Nakamura T., Meyer E., Tasca Gois Ruiz A.L., Foglio M.A., De Carvalho J.E., Da Costa W.F. (2012). Synthesis, antitumor, antitrypanosomal and antileishmanial activities of benzo[4,5]canthin-6-ones bearing the N′-(substituted benzylidene)-carbohydrazide and N-alkylcarboxamide groups at C-2. Chem. Pharm. Bull..

[24] Yang M.L., Kuo P.C., Hwang T.L., Chiou W.F., Qian K., Lai C.Y., Lee K.H., Wu T.S. (2011). Synthesis, in vitro anti-inflammatory and cytotoxic evaluation, and mechanism of action studies of 1-benzoyl-β-carboline and 1-benzoyl-3-carboxy-β-carboline derivatives. Bioorganic Med. Chem..

[25] Guan H., Chen H., Peng W., Ma Y., Cao R., Liu X., Xu A. (2006). Design of β-carboline derivatives as DNA-targeting antitumor agents. Eur. J. Med. Chem..

[26] Funayama Y., Nishio K., Wakabayashi K., Nagao M., Shimoi K., Ohira T., Hasegawa S., Saijo N. (1996). Effects of β- and γ-carboline derivatives on DNA topoisomerase activities. Mutat. Res. Fundam. Mol. Mech. Mutagen..

[27] Song Y., Kesuma D., Wang J., Deng Y., Duan J., Wang J.H., Qi R.Z. (2004). Specific inhibition of cyclin-dependent kinases and cell proliferation by harmine. Biochem. Biophys. Res. Commun..

[28] Castro A.C., Dang L.C., Soucy F., Grenier L., Mazdiyasni H., Hottelet M., Parent L., Pien C., Palombella V., Adams J. (2003). Novel IKK inhibitors: β-carbolines. Bioorg. Med. Chem. Lett..

[29] Sears P.G., Lester G.R., Dawson L.R. (1956). A study of the conductance behavior of some uni-univalent electrolytes in dimethyl sulfoxide at 25°. J. Phys. Chem..

[30] Patra A.K., Bhowmick T., Ramakumar S., Nethaji M., Chakravarty A.R. (2008). DNA cleavage in red light promoted by copper(II) complexes of α-amino acids and photoactive phenanthroline bases. Dalton Trans..

[31] Banaspati A., Das D., Choudhury C.J., Bhattacharyya A., Goswami T.K. (2019). Photocytotoxic copper(II) complexes of N-salicylyl-L-tryptophan and phenanthroline bases. J. Inorg. Biochem..

[32] Ahmad Khan R., Al-Farhan K., De Almeida A., Alsalme A., Casini A., Ghazzali M., Reedijk J. (2014). Light-stable bis(norharmane)silver(I) compounds: Synthesis, characterization and antiproliferative effects in cancer cells. J. Inorg. Biochem..

[33] Khan R.A., de Almeida A., Al-Farhan K., Alsalme A., Casini A., Ghazzali M., Reedijk J. (2016). Transition-metal norharmane compounds as possible cytotoxic agents: New insights based on a coordination chemistry perspective. J. Inorg. Biochem..

[34] Mahato S., Meheta N., Kotakonda M., Joshi M., Shit M., Choudhury A.R., Biswas B. (2020). Synthesis, structure, polyphenol oxidase mimicking and bactericidal activity of a zinc-schiff base complex. Polyhedron.

[35] Amaral T.C., Miguel F.B., Couri M.R.C., Corbi P.P., Carvalho M.A., Campos D.L., Pavan F.R., Cuin A. (2018). Silver(I) and zinc(II) complexes with symmetrical cinnamaldehyde Schiff base derivative: Spectroscopic, powder diffraction characterization, and antimycobacterial studies. Polyhedron.

[36] Maxim C., Pasatoiu T.D., Kravtsov V.C., Shova S., Muryn C.A., Winpenny R.E.P., Tuna F., Andruh M. (2008). Copper(II) and zinc(II) complexes with Schiff-base ligands derived from salicylaldehyde and 3-methoxysalicylaldehyde: Synthesis, crystal structures, magnetic and luminescence properties. Inorg. Chim. Acta.

[37] Khan R.A., Khan M.R., Usman M., Sayeed F., Alghamdi H.A., Alrumman S., Alharbi W., Farshori N.N., Al-Oqail M.M., Siddiqui M.R. (2020). β-Carboline copper complex as a potential mitochondrial-targeted anticancer chemotherapeutic agent: Favorable attenuation of human breast cancer MCF7 cells via apoptosis. Saudi J. Biol. Sci..

[38] Chen L.M., Peng F., Li G.D., Jie X.M., Cai K.R., Cai C., Zhong Y., Zeng H., Li W., Zhang Z. (2016). The studies on the cytotoxicity in vitro, cellular uptake, cell cycle arrest and apoptosis-inducing properties of ruthenium methylimidazole complex [Ru(MeIm)4(p-cpip)]2+. J. Inorg. Biochem..

[39] Karmakar S., Purkait K., Chatterjee S., Mukherjee A. (2016). Anticancer activity of a cis-dichloridoplatinum(ii) complex of a chelating nitrogen mustard: Insight into unusual guanine binding mode and low deactivation by glutathione. Dalton Trans..

[40] Krȩzel A., Bal W. (1999). Coordination chemistry of glutathione. Acta Biochim. Pol..

[41] Florea A.M., Büsselberg D. (2011). Cisplatin as an anti-tumor drug: Cellular mechanisms of activity, drug resistance and induced side effects. Cancers.

[42] Cadoni E., Valletta E., Caddeo G., Isaia F., Cabiddu M.G., Vascellari S., Pivetta T. (2017). Competitive reactions among glutathione, cisplatin and copper-phenanthroline complexes. J. Inorg. Biochem..

[43] Cotgreave I.A., Moldeus P., Orrenius S. (1988). Host biochemical defense mechanisms against prooxidants. Annu. Rev. Pharmacol. Toxicol..

[44] Ortega A.L., Mena S., Estrela J.M. (2011). Glutathione in cancer cell death. Cancers.

[45] Masella R., Di Benedetto R., Varì R., Filesi C., Giovannini C. (2005). Novel mechanisms of natural antioxidant compounds in biological systems: Involvement of glutathione and glutathione-related enzymes. J. Nutr. Biochem..

[46] Gomes A., Fernandes E., Lima J.L.F.C. (2005). Fluorescence probes used for detection of reactive oxygen species. J. Biochem. Biophys. Methods.

[47] Tisato F., Marzano C., Porchia M., Pellei M., Santini C. (2010). Copper in diseases and treatments, and copper-based anticancer strategies. Med. Res. Rev..

[48] Zimmermann T., Burda J.V. (2010). Cisplatin interaction with amino acids cysteine and methionine from gas phase to solutions with constant pH. Interdiscip. Sci. Comput. Life Sci..

[49] Sajid M., Khan M.R., Shah N.A., Ullah S., Younis T., Majid M., Ahmad B., Nigussie D. (2016). Proficiencies of Artemisia scoparia against CCl4 induced DNA damages and renal toxicity in rat. BMC Complement. Altern. Med..

[50] Ebaid H., Habila M., Hassan I., Al-Tamimi J., Omar M.S., Rady A., Alhazza I.M. (2020). Curcumin-containing Silver Nanoparticles prevent carbon tetrachlorideinduced hepatotoxicity in mice. Comb. Chem. High. Throughput Screen..

[51] Ebaid H., Al-Tamimi J., Hassan I., Alhazza I., Al-Khalifa M. (2014). Antioxidant bioactivity of samsum Ant (*Pachycondyla sennaarensis*) Venom protects against CCL4-induced nephrotoxicity in mice. Oxid. Med. Cell. Longev..

[52] Hassan I., Husain F.M., Khan R.A., Ebaid H., Al-Tamimi J., Alhazza I.M., Aman S., Ibrahim K.E. (2019). Ameliorative effect of zinc oxide nanoparticles against potassium bromate-mediated toxicity in Swiss albino rats. Environ. Sci. Pollut. Res..

[53] Das R.K., Hossain S.U., Bhattacharya S. (2007). Protective effect of diphenylmethyl selenocyanate against CCl 4-induced hepatic injury. J. Appl. Toxicol..

[54] Zhong-Feng W., Mao-Yu W., De-Hai Y., Zhao Y., Hong-Mei X., Zhong S., Wen-Yi S., Yu-Fang H., Jun-Qi N., Pu-Jun G. (2018). Therapeutic effect of chitosan on CCl4-induced hepatic fibrosis in rats. Mol. Med. Rep..

[55] Boll M., Weber L.W.D., Becker E., Stampfl A. (2001). Mechanism of carbon tetrachloride-induced hepatotoxicity. Hepatocellular damage by reactive carbon tetrachloride metabolites. Zeitschrift Naturforsch. Sect. C J. Biosci..

[56] Lee C., Yang W., Parr R.G. (1988). Becke’s Three Parameter Hybrid Method Using the LYP. Phys. Rev. B.

[57] Lee C., Yang W., Parr R.G. (1988). Condens. Matter Mater. Phys. Phys. Rev. B.

[58] Stephens P.J., Devlin F.J., Chabalowski C.F., Frisch M.J. (1994). Ab Initio calculation of vibrational absorption and circular dichroism spectra using density functional force fields. J. Phys. Chem..

[59] Frisch M.J. (2009). Gaussian 09.

[60] Hay P.J., Wadt W.R. (1985). Ab initio effective core potentials for molecular calculations. Potentials for the transition metal atoms Sc to Hg. J. Chem. Phys..

[61] Wadt W.R., Hay P.J. (1985). Ab initio effective core potentials for molecular calculations. Potentials for main group elements Na to Bi. J. Chem. Phys..

[62] Roy L.E., Hay P.J., Martin R.L. (2008). Revised basis sets for the LANL effective core potentials. J. Chem. Theory Comput..

[63] Barone V., Cossi M. (1998). Quantum calculation of molecular energies and energy gradients in solution by a conductor solvent model. J. Phys. Chem. A.

[64] Cossi M., Barone V. (2001). Time-dependent density functional theory for molecules in liquid solutions. J. Chem. Phys..

[65] Cossi M., Rega N., Scalmani G., Barone V. (2003). Energies, structures, and electronic properties of molecules in solution with the C-PCM solvation model. J. Comput. Chem..

[66] Badawi A.M., Mohamed M.A.S., Mohamed M.Z., Khowdairy M.M. (2007). Surface and antitumor activity of some novel metal-based cationic surfactants. J. Cancer Res. Ther..

[67] Nagaraj K., Velmurugan G., Sakthinathan S., Venuvanalingam P., Arunachalam S. (2014). Influence of self-assembly on intercalative DNA binding interaction of double-chain surfactant Co(iii) complexes containing imidazo[4,5-f][1,10]phenanthroline and dipyrido[3,2-d:2′-3′-f]quinoxaline ligands: Experimental and theoretical study. Dalton Trans..

[68] Sharifi N., Aragon-Ching J.B. (2009). Anti-Cancer Agents in Medicinal Chemistry: Editorial. Anticancer Agents Med. Chem..

[69] Shao J., Ma Z.Y., Li A., Liu Y.H., Xie C.Z., Qiang Z.Y., Xu J.Y. (2014). Thiosemicarbazone Cu(II) and Zn(II) complexes as potential anticancer agents: Syntheses, crystal structure, DNA cleavage, cytotoxicity and apoptosis induction activity. J. Inorg. Biochem..

[70] Kelkel M., Jacob C., Dicato M., Diederich M. (2010). Potential of the dietary antioxidants resveratrol and curcumin in prevention and treatment of hematologic malignancies. Molecules.

[71] Linden A., Gülden M., Martin H.J., Maser E., Seibert H. (2008). Peroxide-induced cell death and lipid peroxidation in C6 glioma cells. Toxicol. Vitr..

[72] Draper H.H., Hadley M. (1990). Malondialdehyde determination as index of lipid Peroxidation. Methods Enzymol..

[73] Siddiqui M.A., Singh G., Kashyap M.P., Khanna V.K., Yadav S., Chandra D., Pant A.B. (2008). Influence of cytotoxic doses of 4-hydroxynonenal on selected neurotransmitter receptors in PC-12 cells. Toxicol. Vitr..

[74] Siddiqui M.A., Kashyap M.P., Kumar V., Al-Khedhairy A.A., Musarrat J., Pant A.B. (2010). Protective potential of trans-resveratrol against 4-hydroxynonenal induced damage in PC12 cells. Toxicol. In Vitro.

[75] Siddiqui M.A., Ahmad J., Farshori N.N., Saquib Q., Jahan S., Kashyap M.P., Ahamed M., Musarrat J., Al-Khedhairy A.A. (2013). Rotenone-induced oxidative stress and apoptosis in human liver HepG2 cells. Mol. Cell. Biochem..

[76] Chandra D., Ramana K.V., Wang L., Christensen B.N., Bhatnagar A., Srivastava S.K. (2002). Inhibition of fiber cell globulization and hyperglycemia-induced lens opacification by aminopeptidase inhibitor bestatin. Investig. Ophthalmol. Vis. Sci..

[77] Buege J.A., Aust S.D. (1978). Microsomal lipid peroxidation. Methods in Enzymology.

[78] Tabassum S., Asim A., Khan R.A., Hussain Z., Srivastav S., Srikrishna S., Arjmand F. (2013). Chiral heterobimetallic complexes targeting human DNA-topoisomerase Iα. Dalton Trans..

[79] Khan R.A., Yadav S., Hussain Z., Arjmand F., Tabassum S. (2014). Carbohydrate linked organotin(iv) complexes as human topoisomerase Iα inhibitor and their antiproliferative effects against the human carcinoma cell line. Dalton Trans..

[80] Yousuf I., Arjmand F., Tabassum S., Toupet L., Khan R.A., Siddiqui M.A. (2015). Mechanistic insights into a novel chromone-appended Cu(II) anticancer drug entity: In vitro binding profile with DNA/RNA substrates and cytotoxic activity against MCF-7 and HepG2 cancer cells. Dalton Trans..

[81] Tabassum S., Asim A., Khan R.A., Arjmand F., Rajakumar D., Balaji P., Akbarsha M.A. (2015). A multifunctional molecular entity CuII-SnIV heterobimetallic complex as a potential cancer chemotherapeutic agent: DNA binding/cleavage, SOD mimetic, topoisomerase Iα inhibitory and in vitro cytotoxic activities. RSC Adv..

[82] Hassan I., Ebaid H., Alhazza I.M., Al-Tamimi J., Aman S., Abdel-Mageed A.M. (2019). Copper mediates anti-inflammatory and antifibrotic activity of gleevec in hepatocellular carcinoma-induced male rats. Can. J. Gastroenterol. Hepatol..

[83] Alhazza I.M., Hassan I., Ebaid H., Al-Tamimi J., Alwasel S.H. (2020). Chemopreventive effect of riboflavin on the potassium bromate–induced renal toxicity in vivo. Naunyn. Schmiedebergs. Arch. Pharmacol..

[84] Jollow D., Mitchell J.R., Zampaglione N., Gillette J.R. (1974). Bromobenzene-induced liver necrosis. Protective role of glutathione and evidence for 3,4-bromobenzene oxide as the hepatotoxic metabolite. Pharmacology.

[85] Singh N.P., McCoy M.T., Tice R.R., Schneider E.L. (1988). A simple technique for quantitation of low levels of DNA damage in individual cells. Exp. Cell Res..

[86] Hassan I., Chibber S., Khan A.A., Naseem I. (2012). Riboflavin ameliorates cisplatin induced toxicities under photoillumination. PLoS ONE.

