# Supplementary material for: Bioactive Tryptophan-Based Copper Complex with Auxiliary β-Carboline Spectacle Potential on Human Breast Cancer Cells: In Vitro and In Vivo Studies
Source: Molecules. 2021 Mar 14;26(6):1606. doi: 10.3390/molecules26061606 (PMC8001361; doi:10.3390/molecules26061606)
Supplement: Supplementary file 1 [file molecules-26-01606-s001.pdf]

Supporting Information

Bioactive tryptophan-based copper complex with auxiliary β-carboline spectacle potential on human breast cancer cells: *In vitro* and *in vivo* studies

Walaa AlHarbi,[a,1,*] Iftekhar Hassan,[b,1] Rais Ahmad Khan,[c,1,*] Shazia Parveen,[d] K. H. Alharbi,[e] Ibtisam I BinSharfan,[c] Ibrahim M. Alhazza,[b] Hossam Ebaid,[b] Ali Alsalme.[c]

[a]*Department of Chemistry, Faculty of Science, King Khalid University, P.O. Box-9004, Abha 62529, KSA.*
[b]*Department of Zoology, College of Science, King Saud University, P.O. Box 2455, Riyadh 11451, KSA.*
[c]*Department of Chemistry, College of Science, King Saud University, P.O. Box 2455, Riyadh 11451, KSA.*
[d]*Chemistry Department, Faculty of Science, Taibah University, Yanbu Branch, 46423, Yanbu, KSA.*
[e]*Department of Chemistry, Science and Arts College, Rabigh Campus, King Abdulaziz University, Jeddah 21911, Saudi Arabia*

[1]*Authors contributed equally.*

**Author for Correspondence: krais@ksu.edu.sa (Khan, R.A.); Mobile +966 536745404 and co-correspondence wal-harbe@kku.edu.sa (AlHarbi, W.)*

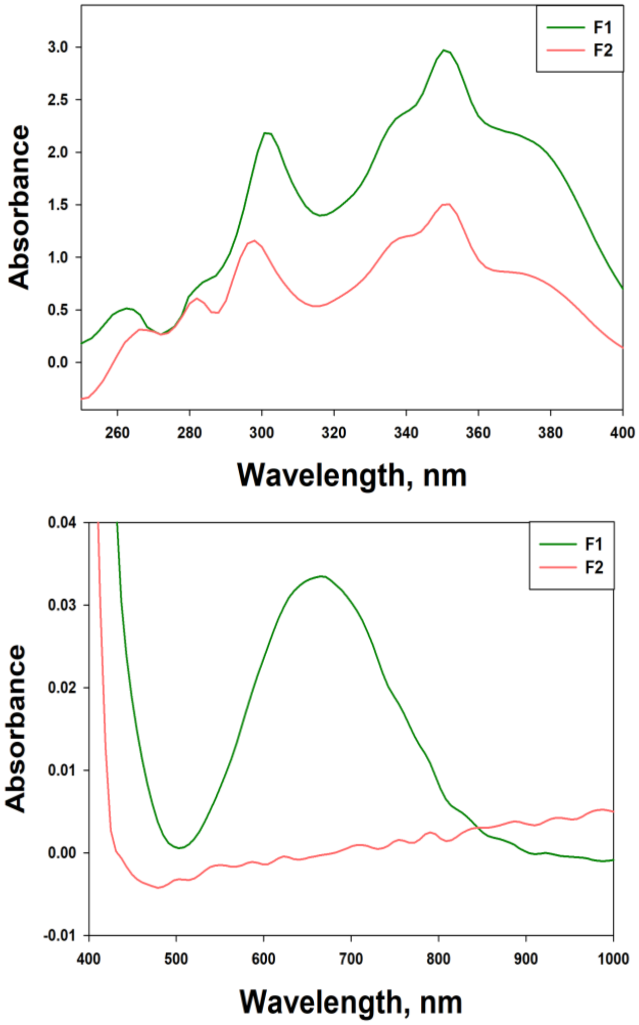

Figure S1. Absorbance spectra of complexes **1** and **2**.

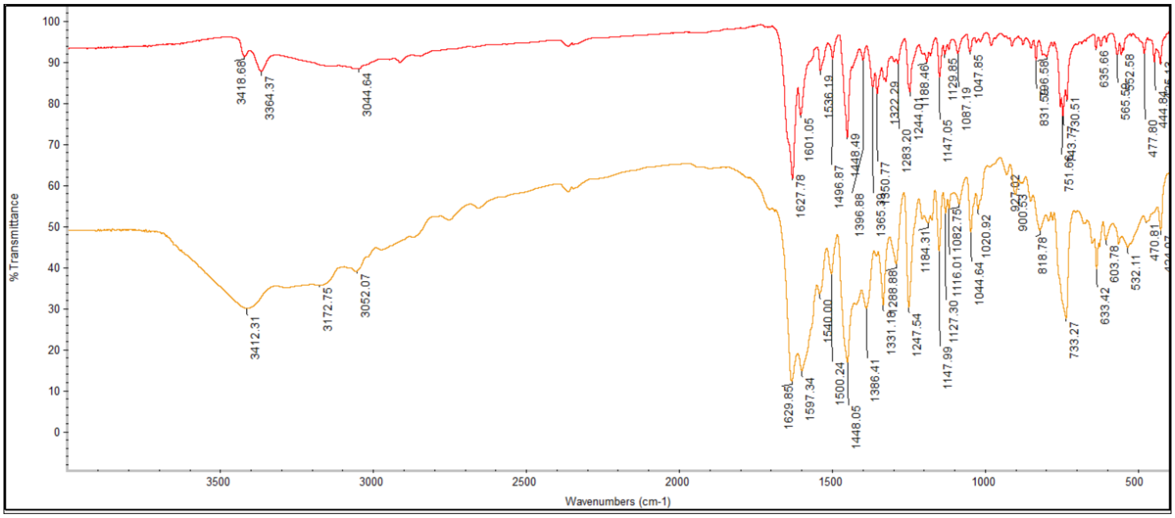

Figure S2. IR spectra of complexes **1** and **2**.

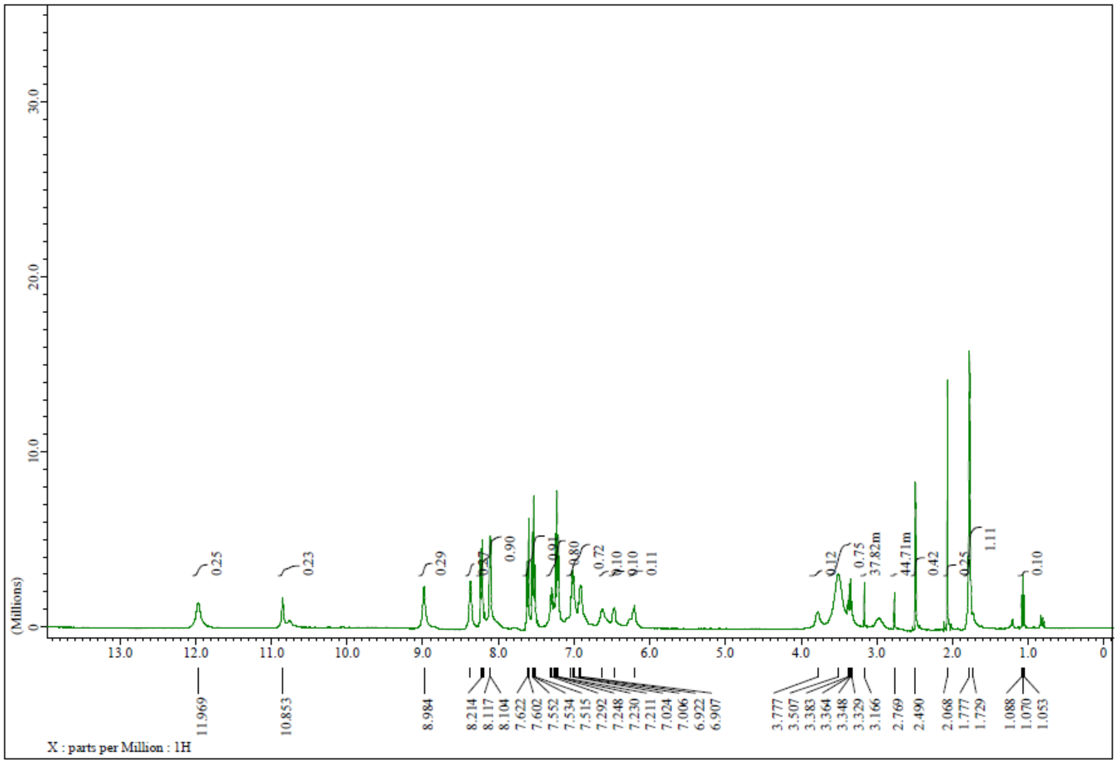

Figure S3. ^{1}H NMR of Zn complex, **2.**

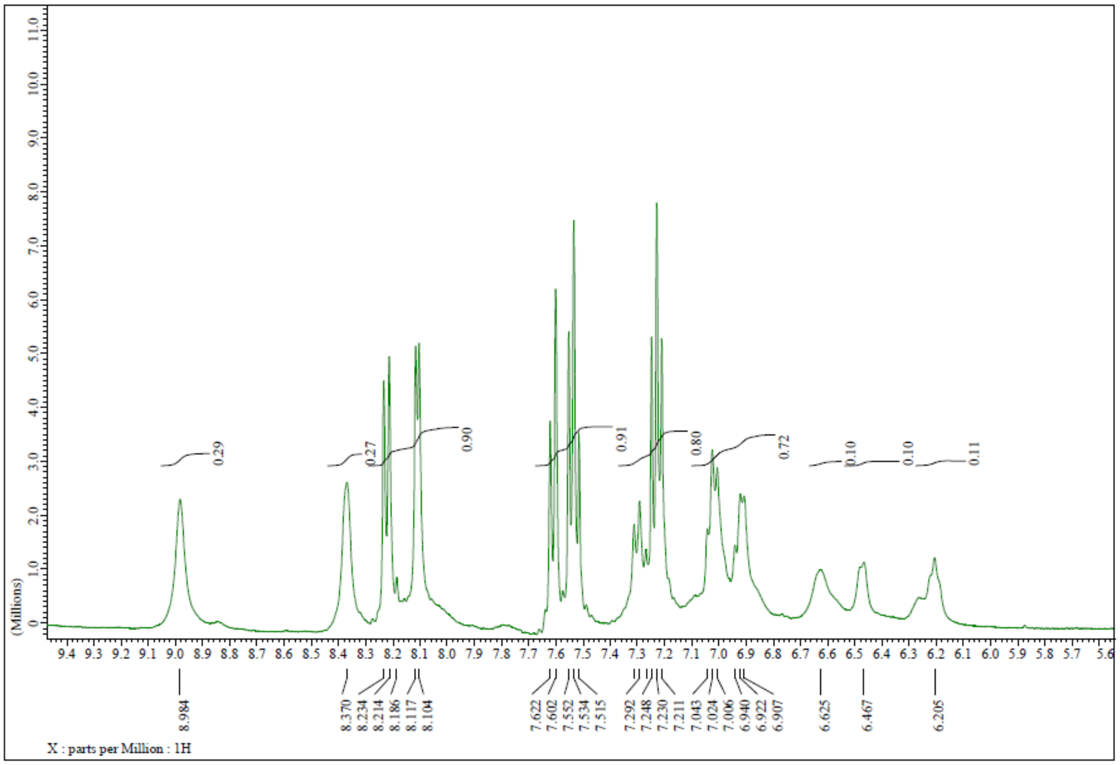

Figure S4. ^{1}H NMR of Zn complex, **2** (aromatic region).

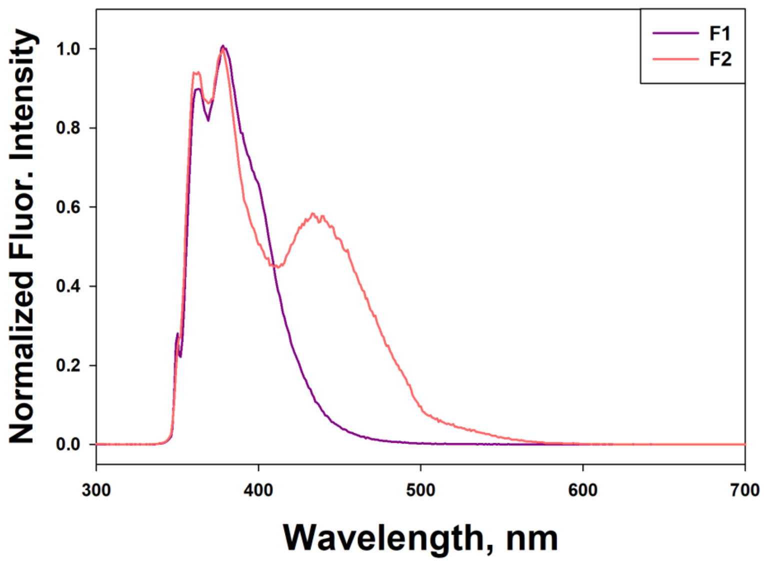

Figure S5. Fluorescence spectra of complexes **1** and **2**.

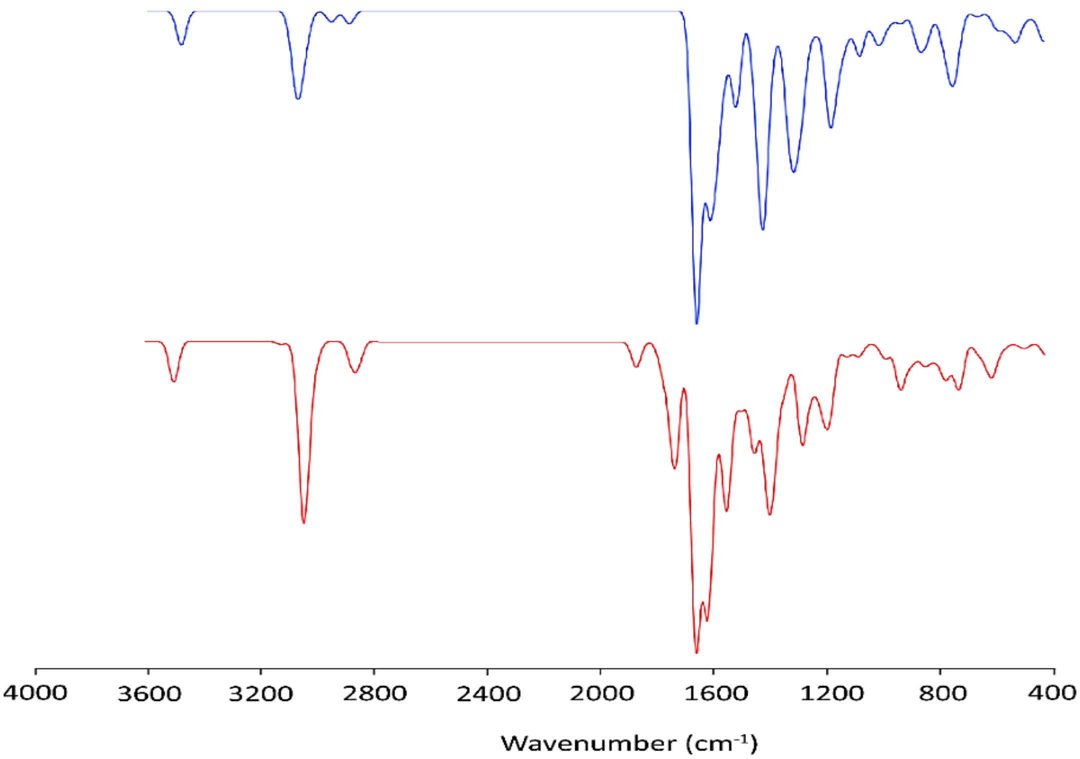

Figure S6. DFT calculated vibrational spectra of complex **1** (blue) and **2** (red).

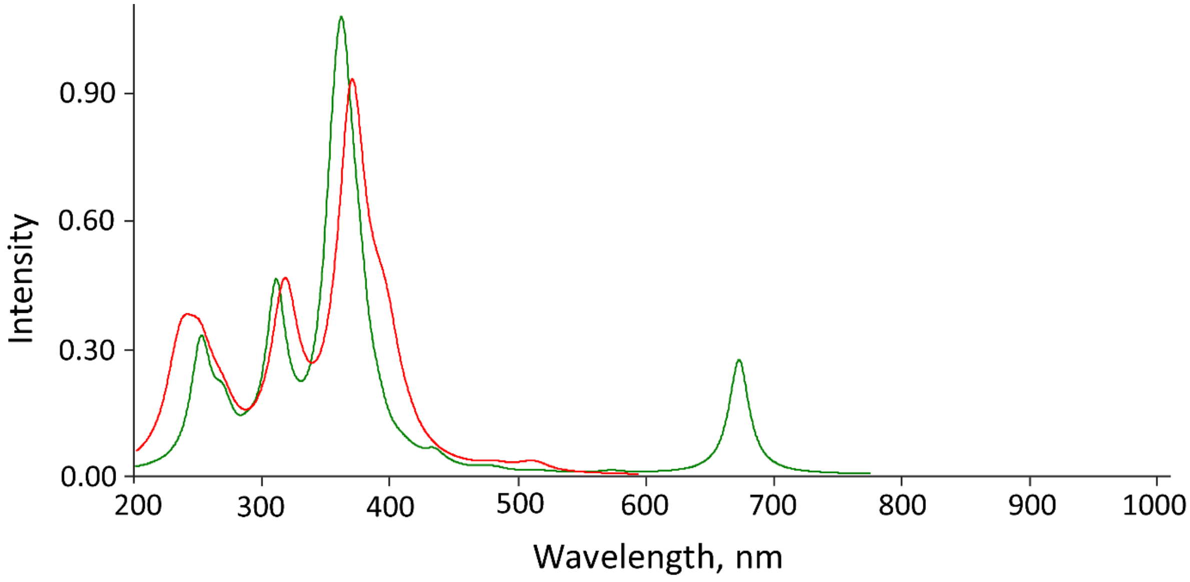

Figure S7. TDDFT calculated electronic absorption spectra of complex **1** (red) and **2** (blue).

Table S1. The Cartesian atomic coordinates of the calculated optimized structures **1** and **2** in DMSO.

Cu(II) complex **1**				Zn(II) complex **2**			
C	7.927081000	-0.690136000	-1.489278000	C	-7.676576000	1.646261000	-1.389665000
C	7.323464000	-1.961183000	-1.610551000	C	-6.866470000	2.765350000	-1.659763000
C	6.024162000	-2.186755000	-1.170106000	C	-5.559964000	2.844386000	-1.186425000
C	5.307658000	-1.124642000	-0.586154000	C	-5.052500000	1.777524000	-0.422246000
C	5.935247000	0.149772000	-0.478036000	C	-5.887087000	0.655479000	-0.167400000
C	7.242287000	0.381215000	-0.925275000	C	-7.198201000	0.572765000	-0.640186000
C	3.989790000	-0.993001000	-0.009993000	C	-3.790316000	1.523112000	0.233859000
C	3.870710000	0.314762000	0.401871000	C	-3.902604000	0.282182000	0.831648000
N	5.032073000	1.002560000	0.119942000	N	-5.153739000	-0.237084000	0.595341000
C	2.952503000	-2.085891000	0.132596000	C	-2.586549000	2.426703000	0.274256000
N	1.857252000	-1.687910000	1.023732000	N	-1.515712000	1.847546000	1.077460000
C	2.346435000	-2.453686000	-1.250030000	C	-2.043265000	2.643576000	-1.202253000
O	3.022703000	-3.106940000	-2.046374000	O	-2.562550000	3.538357000	-1.859603000
O	1.147765000	-2.014865000	-1.480897000	O	-1.117120000	1.828386000	-1.566419000
C	2.014647000	-1.733306000	2.306333000	C	-1.406714000	2.167465000	2.325506000
C	1.034203000	-1.371993000	3.295326000	C	-0.416826000	1.669532000	3.251084000
C	1.409367000	-1.491787000	4.656892000	C	-0.510806000	2.113493000	4.589064000
C	0.546932000	-1.158620000	5.683944000	C	0.362237000	1.680809000	5.571633000
C	-0.740202000	-0.686273000	5.357639000	C	1.377232000	0.764873000	5.224838000
C	-1.141980000	-0.558397000	4.039576000	C	1.506478000	0.308700000	3.929028000
C	-0.279857000	-0.895511000	2.957582000	C	0.629688000	0.738030000	2.883495000
O	-0.725326000	-0.751645000	1.737454000	O	0.808964000	0.285696000	1.685328000
C	0.825248000	1.230519000	-1.889715000	Zn	-0.073744000	0.809241000	-0.110720000
N	0.254007000	0.943389000	-0.710572000	C	-1.339517000	-1.399769000	-1.762633000
C	-0.297400000	1.940099000	0.021723000	N	-0.510584000	-1.190092000	-0.716848000
C	-0.280490000	3.248862000	-0.423127000	C	0.010114000	-2.250955000	-0.047073000
C	0.327918000	3.548018000	-1.676427000	C	-0.293731000	-3.544442000	-0.416716000
C	0.892996000	2.523219000	-2.429924000	C	-1.176829000	-3.772131000	-1.526701000

C	-0.758333000	4.535852000	0.078707000	C	-1.704918000	-2.671348000	-2.208577000
C	-0.379246000	5.480268000	-0.943329000	C	0.058802000	-4.874253000	0.048826000
N	0.267214000	4.905006000	-1.991585000	C	-0.657195000	-5.758894000	-0.842447000
C	-1.423905000	4.962546000	1.211552000	N	-1.391272000	-5.105023000	-1.777703000
C	-1.719137000	6.338001000	1.336924000	C	0.853013000	-5.391301000	1.061522000
C	-1.353770000	7.266224000	0.345913000	C	0.943941000	-6.786629000	1.196456000
C	-0.682666000	6.853939000	-0.801501000	C	0.250118000	-7.657406000	0.331263000
C	-1.745154000	-2.189390000	-2.086621000	C	-0.550327000	-7.162217000	-0.688135000
N	-1.701167000	-1.671442000	-0.845586000	C	1.872218000	2.069940000	-2.118848000
C	-2.856784000	-1.384355000	-0.193710000	N	1.830099000	1.360765000	-0.970523000
C	-4.079751000	-1.615209000	-0.790037000	C	2.991617000	0.983714000	-0.370800000
C	-4.126155000	-2.163285000	-2.104285000	C	4.213630000	1.308417000	-0.918539000
C	-2.938898000	-2.457844000	-2.767496000	C	4.262097000	2.061239000	-2.138622000
C	-5.479122000	-1.436241000	-0.408346000	C	3.063562000	2.450300000	-2.742586000
C	-6.222062000	-1.903280000	-1.553485000	C	5.608285000	1.083306000	-0.570852000
N	-5.428864000	-2.337525000	-2.566923000	C	6.342977000	1.730522000	-1.633946000
C	-6.140219000	-0.961573000	0.707273000	N	5.544976000	2.308978000	-2.565738000
C	-7.552756000	-0.947395000	0.692524000	C	6.282313000	0.446820000	0.458297000
C	-8.286242000	-1.400156000	-0.418816000	C	7.688993000	0.446088000	0.439892000
C	-7.636625000	-1.881738000	-1.550843000	C	8.413702000	1.073260000	-0.592596000
H	8.943162000	-0.544723000	-1.846200000	C	7.758268000	1.717414000	-1.633268000
H	7.885187000	-2.774054000	-2.063339000	H	-8.692318000	1.614645000	-1.774125000
H	5.550257000	-3.154519000	-1.296282000	H	-7.268159000	3.580020000	-2.255619000
H	7.702318000	1.361243000	-0.833333000	H	-4.922345000	3.689212000	-1.421881000
H	3.044437000	0.813740000	0.885970000	H	-7.822763000	-0.292201000	-0.432755000
H	5.197930000	1.972634000	0.343735000	H	-3.180921000	-0.262020000	1.422378000
H	3.438515000	-2.992463000	0.519591000	H	-5.481345000	-1.131274000	0.926551000
H	2.981386000	-2.071970000	2.695317000	H	-2.874994000	3.418594000	0.648278000
H	2.409246000	-1.856067000	4.883245000	H	-2.133748000	2.871000000	2.753940000
H	0.853781000	-1.257069000	6.720323000	H	-1.302092000	2.816574000	4.842642000
H	-1.432272000	-0.417785000	6.152367000	H	0.267602000	2.039374000	6.591731000
H	-2.135060000	-0.194393000	3.791433000	H	2.067949000	0.414611000	5.988353000
H	1.244940000	0.382172000	-2.422033000	H	2.286366000	-0.396188000	3.655250000
H	-0.745780000	1.635774000	0.963139000	H	-1.711094000	-0.500741000	-2.244639000
H	1.365066000	2.706580000	-3.389180000	H	0.658165000	-1.999759000	0.787736000
H	-1.719007000	4.270226000	1.994474000	H	-2.375179000	-2.795441000	-3.052331000
H	-2.242075000	6.685524000	2.222858000	H	1.398114000	-4.741992000	1.741669000
H	-1.599792000	8.314642000	0.481481000	H	1.563496000	-7.204403000	1.985067000
H	-0.393037000	7.556530000	-1.576535000	H	0.348063000	-8.730361000	0.469049000
H	-0.779859000	-2.391393000	-2.536041000	H	-1.090427000	-7.818095000	-1.363702000
H	-2.736983000	-0.974779000	0.803049000	H	0.902378000	2.328864000	-2.531890000
H	-2.925296000	-2.876634000	-3.767733000	H	2.873518000	0.429552000	0.555064000
H	-5.602855000	-0.605293000	1.581029000	H	3.051967000	3.024740000	-3.662521000
H	-8.084248000	-0.576588000	1.563710000	H	5.748766000	-0.044374000	1.267679000
H	-9.370754000	-1.371767000	-0.388204000	H	8.228949000	-0.050384000	1.241292000
H	-8.186084000	-2.235349000	-2.417480000	H	9.499254000	1.049614000	-0.569061000
Cu	0.162509000	-1.267070000	0.070151000	H	8.299072000	2.206578000	-2.437254000
